# Attractiveness and Sexual Competitiveness of *Anastrepha obliqua* Males (Diptera: Tephritidae) Fed on a Diet Enriched With *Providencia rettgeri*

**DOI:** 10.3389/fmicb.2020.01777

**Published:** 2020-07-28

**Authors:** Linnet Roque-Romero, Emilio Hernández, Marysol Aceituno-Medina, Carmen Ventura, Jorge Toledo, Edi A. Malo

**Affiliations:** ^1^Instituto de Biociencias, Universidad Autónoma de Chiapas, Chiapas, Mexico; ^2^Programa Moscafrut SADER-SENASICA, Subdirección de Desarrollo de Métodos, Chiapas, Mexico; ^3^Grupo de Ecología de Artrópodos y Manejo de Plagas, El Colegio de la Frontera Sur, Chiapas, Mexico

**Keywords:** sterile insect technique, sexual competitiveness, field cages, pheromone components, diet

## Abstract

The West Indian fruit fly, *Anastrepha obliqua* (Macquart), is the second most important tephritid fruit fly in Mexico, infesting mango, hog plum and guava fruits. To control this pest, the Mexican government has implemented the use of the sterile insect technique (SIT), which involves the mass production, sterilization and release of flies. However, the *A. obliqua* laboratory males used in SIT are selected to a lesser extent by the wild females during competitiveness tests. The objective of this study was to compare the effects of males fed on fruit fly food enriched with *Providencia rettgeri* to those in males fed on food alone, assessing male mating competitiveness, capture of females using traps baited with males fed with the enriched diet and sex pheromone components. The results indicated that males fed with the diet enriched with *P. rettgeri* had increased mating competitiveness and captured more females in the field cage tests. However, no difference was observed in the proportion of volatile sex pheromone components identified during the calling of *A. obliqua* males. The results suggest the value of incorporating bacteria into the mass rearing technique of *A. obliqua* adults in order to improve the sexual competitiveness of males from the laboratory compared to wild males.

## Introduction

An important aspect of insect biology that allows these organisms to increase their diversity and abundance is the symbiotic relationships they share with microorganisms that impact directly on their life history traits. Although many of these microorganisms may be commensalist or parasitic (Poveda-Arias, 2019), others play an important role in the nutrition, metabolism and immune protection of their hosts (Dillon and Dillon, 2004; Brune, 2010; Koch and Schmid-Hempel, 2011; Engel and Moran, 2013). Early studies in the Mexican fruit fly, *Anastrepha ludens* (Loew) were carried out to evaluate resistance or sensitivity to a variety of antibiotics in bacteria isolated from this same species (Kuzina et al., 2001) and to test the attractive effect of metabolites produced by bacteria on *A. ludens* (Martínez et al., 1994). In addition, studies have determined the presence of *Wolbachia* in *Anastrepha* species (Mateos et al., 2020), including guava fruit fly, *A. striata* (Martínez et al., 2012), West Indian fruit fly, *A. obliqua* (Mascarenhas et al., 2016), South American fruit fly, *A. fraterculus* (Conte et al., 2019) and sapote fruit fruit fly, *A. serpentina* (Coscrato et al., 2009). On the other hand, studies have revealed that the gut bacterial community in flies consists mainly of species of the Enterobacteriaceae family (Noman et al., 2020), and that these microorganisms may be involved in nitrogen fixation, nutrition and insect fitness, including mating behavior and sexual competitiveness, reproductive success, longevity, improve the males fitnnes (Gavriel et al., 2011; Hamden et al., 2013; Andongma et al., 2015; Khaeso et al., 2017; Yuval, 2017; Juárez et al., 2019), protection against pathogens and detoxification (Ben-Yosef et al., 2015; Guo et al., 2017).

Some of the species of the Enterobacteriaceae family correspond to *Klebsiella*, *Enterobacter*, *Providencia*, *Pectobacterium*, *Pantoea*, *Morganella* and *Citrobacter* (Ben-Ami et al., 2010; Aharon et al., 2013). *Providencia rettgeri* is a gram negative, opportunistic pathogenic bacteria for humans (O’Hara et al., 2000), which has also been reported as pathogenic for *Drosophila melanogaster* (Galac and Lazzaro, 2011). However, the pathogenicity of *P. rettgeri* in Mediterranean fruit fly, *Ceratitis capitata* (Wied.) depends on the concentration supplied (Msaad-Guerfali et al., 2018), and previous experiments indicate that *A. obliqua* males can actually increase their sexual competitiveness under laboratory conditions when fed on *P. rettgeri* (Gómez-Alonso, 2013).

The West Indian fruit fly, *A. obliqua* (Macquart), is considered one of the most important tephritid fruit flies affecting the fruit production industry in Mexico and many tropical countries of America because it infests mangoes (*Mangifera indica* L.), hog plum fruits (*Spondias* spp.), sapodilla (*Achras zapota* L.), carambola (*Averrhoa carambola* L., Oxalidaceae) and guava (*Psidium guajava* L.) (Norrbom and Kim, 1988; Hernández-Ortiz, 1992; Birke et al., 2013; Aluja et al., 2014). To avoid or minimize the harmful effects of *A. obliqua*, mango growers must comply with the health and safety standards required by the market, applying an area-wide management approach involving chemical, biological, cultural and sterile insect (SIT) techniques (Reyes et al., 2000). The SIT involves the mass production of 70 million of bisexual pupae of the target species per week, followed by their sterilization and release (Reyes et al., 2000; Domínguez et al., 2010). However, the *A. obliqua* males used in the SIT are selected for mating by the wild females with a lower frequency (Rull et al., 2012) and, according to that observed for *Ceratitis capitata* by Lance et al. (2000), could therefore be considered less competitive than the wild males.

The sexual competitiveness of sterile male fruit flies is increased by using different strategies: 1) increasing the protein content in the diet provided during sexual maturation (Yuval et al., 2007); 2) adding the juvenile hormone analog (methoprene) to the adult diets (Pereira et al., 2011; Teal et al., 2013; Muñoz-Barrios et al., 2016); 3) applying aromatherapy by exposing the males to volatiles of ginger (*Zingiber* officinale Roscoe) (Shelly, 2006; Flores et al., 2011) and orange (*Citrus sinensis* L.) (Charalampos et al., 2012; Segura et al., 2018) oil; and 4) using enriched foods containing Enterobacteriaceae (Ben-Ami et al., 2010; Gómez-Alonso, 2013; Yuval et al., 2013; Augustinos et al., 2015). In this way, although *A. obliqua* shows facultative autogeny (Polloni and Da Costa-Telles, 1987), foods with nitrogenous compounds are key for the *A. obliqua* males to complete sexual maturation and increase their sexual competitiveness. However, insects cannot synthesize some of the protein-forming amino acids. Many insects that feed on plant material rely on symbiotic association with microorganisms for some aspect of their nutrition (Fitt and O’Brien, 1985). Particularly in Dipterans, it has been documented that bacteria in the digestive tract can mitigate this metabolic limitation, providing their host with essential amino acids (Boush and Matsumura, 1967; Douglas, 2009). Gut bacteria facilitate the absorption of some nutrients by providing digestive enzymes, e.g., microbial hydrolases (Ben-Yosef et al., 2008). Previous studies under laboratory conditions indicate that the main benefit of food enriched with bacteria is an increase in the incidence of mating in *A. obliqua* when males are fed with *P. rettgeri* (Gómez-Alonso, 2013). The objective of this study was to evaluate under field cage conditions the effect of an diet enriched with *P. rettgeri* on males mating competitiveness, male attractiveness and pheromone components of *A. obliqua*.

## Materials and Methods

This research was performed in the Methods Development laboratory of the Moscafrut Program (SADER-SENASICA) in Metapa de Dominguez, Chiapas.

### Obtaining Insects

Pupae were obtained from the Moscafrut (SADER-SENASICA) facility, located in Metapa de Dominguez, Chiapas, Mexico, from a colony that had been mass-reared for more of 150 generations (Orozco-Dávila et al., 2014). All of the pupae used in this study were irradiated for 48 h pre-emergence with 80 Gy of gamma radiation using a Cobalt 60 source (Toledo et al., 2004). The mass-rearing procedures and conditions followed those described by Artiaga-López et al. (2004). The adults were separated by sex at 2 d of age. In all experiments, we used 100 male flies per treatment, which were placed in separate acrylic cages (30 × 30 × 30 cm). To replace the dead flies, 500 females were placed in acrylic cages (30 × 30 × 40 cm) for the mating tests. Both males and females were kept at a density of 1 fly per cm^3^, at 26 ± 1°C and 70–80% RH under a photoperiod of 12:12 h (L:D). The photophase began at 07:00 h and ended at 19:00 h (FAO, 2007).

### Obtaining Bacteria

The bacteria strain of *P. rettgeri* used in this experiment was previously isolated by Gómez-Alonso (2013) from the guts of wild larvae and adult flies obtained from infested hog plum fruits collected in Metapa de Dominguez, Chiapas and indentified in Laboratory of Microbiology of the Instituto Politécnico Nacional (IPN) in Mexico City. For the purpose of this study, *P. rettgeri* was isolated from adults caught in traps loca ted around of Metapa de Dominguez Chiapas (14°50′N, 92°11′W). A sample of 30 larvae were kept for pupation and adult emergence. Prior to dissection, the insects were superficially disinfected by repeated immersion in solutions of 10% sodium hypochlorite, 70% ethanol (v/v), with a final wash in sterile distilled water. Each of these steps was carried out for 1-min. The larvae were then dissected in sterile conditions under a stereoscopic microscope to obtain the intestines, which were placed in phosphate buffered saline (PBS) and ground using sterile rods. The homogeneous solution of intestinal tracts was then used to make serial dilutions and spread on duplicate plates of nutritive agar (DIBICO, Cuautitlán Izcalli, Mexico City). These plates were incubated at 28°C for 1 to 2 days, and each colony type was then categorized and quantified. Pure cultures were obtained and stored at −70°C for further analysis.

The presumptive identification of the isolates was performed by the Analytical Profile Index (API) (BioMerieux, Hazelwood, Mo) and the results had a >98% accuracy for *P. rettgeri* (Code API 20E: 027431157). Final identification of the strains was performed by sequencing the 16S *rRNA* gene (1500 bp), for which *DNA* was obtained from each isolate using the modified technique described by Hoffman and Winston (1987). The isolated bacteria were grown in 20 ml of nutrient broth (DIBICO) and incubated at 28°C for 24 – 48 h with shaking (120 rpm). The suspension was centrifuged at 8000 rpm, and the resulting cell pellet was subjected to chemical breakdown by adding 500 μl of lysis solution (Tris–HCl 10 mM, pH 8.0; 1 Mm EDTA; NaCl 10 mM; SDS 1% Triton X-100), and mechanical breakdown using glass beads (0.5 g). Next, 200 μL of phenol-chloroform-isoamyl alcohol (25: 24: 1) was added and the mixture agitated for 3 min using a vortex and incubated at −70°C for 20 min before undergoing heat shock at 65°C for 30 min. After the incubation, the sample was centrifuged at 14,000 rpm for 5 min, and the supernatant placed in a new tube. To precipitate the DNA, 1 ml of isopropanol was added, and the sample was incubated at −20°C for 20 min and centrifuged at 14,000 rpm for 10 min. The supernatant was removed and evaporated on a hub (A160 Speedvac) at 45°C, and the resulting pellet suspended in 30 μl of injectable water. DNA integrity was visualized by gel electrophoresis using a 1% agarose gel.

The PCR amplification of the 16S *rRNA* gene was conducted using a fragment of the 16S r*DNA* gene of approximately 1500 bp in length, which was obtained from the *DNA* amplified using the universal primers 27F (5′-AGA GTT TCM TGG CTC AG-3′) and 1492R (5′-TAC GGY TAC CTT ACG ACT T-3′) (Lane, 1991). The reaction mixture had a final volume of 25 μL, and included 2.5 μL of Buffer (10X) containing MgCl_2_ (50 mM), 1.0 μL of dNTPs (10 mM), 1.0 μL of initiator 27 F (0.1 nm/μL), 1.0 initiator 1492R (0.1 nm/μL), 1.0 μL of DNA (>80 ng/μL), and 0.2 μL of Taq DNA polymerase (Invitrogen) (5 U/μL). The amplification conditions were an initial denaturation at 94°C for 5 min, followed by 25 cycles of 94°C for 1 min, 57°C for 1 min, 72°C for 1 min, and a final extension step at 72°C for 5 min. The amplified product was purified using the GFX PCR DNA and Gel Band Purification kit (GE). DNA integrity was verified by electrophoresis using a 1% agarose gel. The amplified sequence was obtained using the ABI Prism 3100 (Applied Biosystems) DNA sequencer.

The isolated 16S *rDNA* gene sequence was compared to the reference sequences deposited in the databases of the National Center for Biotechology Information (NCBI). Alignment of the reference sequences and the query sequence was performed using the ClustalX program v.1.83. Editing and removal of ambiguous sites of the query sequence was performed with the Seaview v.2.01 program. Taxonomic identification of the bacteria was conducted by alignment of the isolated and reference sequences according to the criteria of Rossello-Mora and Amann (2001) and using a 97% similarity of the 16S *rRNA* genes based on the similarity matrix following the Myers and Miller (1988) method, implemented in the program MatGAT v.2.1 (Campanella et al., 2003).

#### Kinetics of *Providencia rettgeri* Growth

The viability of the *P. rettgeri* used in our experiments was ensured obtaining bacteria in the logarithmic growth phase. For this, *P. rettgeri* was grown in 100 ml of nutrient broth (DIBICO) and incubated at 35 ± 2°C for 24–48 h with shaking (120 rpm). The growth kinetics were performed in a culture medium of 100 mL and the absorbance of inoculum of 0.2. The growth of *P. rettgeri* was determined by spectrophotometry at 600 nm (Jenway brand, model 6715, Stone Bibby Scientific Ltd., China) and count by mass dilution. In addition, the absorbance and the quantity of colony forming units (CFU) per ml were correlated. The bacterial concentration was determined by taking 0.2 ml samples of the pure culture to make serial dilutions, of which the dilutions 10^–4^–10^–6^ were seeded in triplicate using the standard plate agar method. The colony forming units (CFU) in the plates with nutrient agar were quantified after 48 h at 36°C. The bacterial growth curve was then calculated and the bacterial concentration estimated at different times of growth.

#### Incorporation of *P. rettgeri* Into the Fly Diet

The logarithmic phase bacterial culture (Abs = 0.8 nm, 720 min) was separated from the liquid medium by centrifugation at 8000 rpm for 10 min. The concentration of microorganisms used was 5 × 10^4^ CFU per gram of food (Gómez-Alonso, 2013). The resulting microorganism pellet was then suspended in 1 ml of sterile distilled water, and this suspension was immediately added to the Mb^®^ food and mixed until completely homogenized.

The Mb^®^ food consisted of a mixture of amaranth flour (*Amaranthus cruentus* L.), refined sugar and peanuts (*Arachis hipogaea* L.). It contained 10.19% protein, had a pH of 6.15 UI (Hernández et al., 2010), and was developed to feed the adults of *Anastrepha* spp. during sexual maturation under confined conditions in the facility, for subsequent release in application of the SIT.

#### Feeding the Flies

Males were fed with diet corresponding to each treatment: Mb^®^ food or Mb^®^ food enriched with *P. rettgeri*. The laboratory females were fed with Mb^®^ enriched with the bacteria. The Mb^®^ food enriched with the bacterium *P. rettgeri* was renewed every two days, since this is the length of time for which the >50% bacterial cells remain viable (living cells capable of reproducing) in the food (Gómez-Alonso, 2013).

### Experimental Design

The evaluation under field cage conditions of the effect of diet enriched with *P. rettgeri* on males mating competitiveness, attractiveness capture and pheromone components of *A. obliqua* was performed according to 3-factors experimental desing for sexual competitiveness and attractiveness. Factor 1: Diet with and without bacteria. Factor 2: Age (8–10 days old) of the males,. Factor 3: Cohort, 1, 2, 3, and 4. The variables reponse corresponded to the percentage of matings to determine the effect of the treatments on the sexual competitiveness, the percentage of flies caught in traps in a field cage to evaluate attractiveness. Three replicates were performed for each treatment. The effect on the pheromone compounds was evaluated in a design in blocks, the factor corresponded to the diet with and without bacteria and the age (8 and 9 days old) of the males was considered as blocks. The area under each peak was determined, and this was the variable analyzed in order to assess the effect of diet on the sex pheromone components. Four replicates were performed for each treatment.

### Sexual Competitiveness Tests in Field Cages

The tests of sexual competitiveness, capture and analysis of volatiles were carried out after the flies were 8 days old, in order to ensure sexual maturity, which was determined by observing the characteristics of this phase: vigorous fluttering and dilatation of the pleural glands (males) and the presence of eggs (females) (Aluja, 1993).

This test was performed in a field cage of 3 m in diameter and 2 m in height, supported by a metal structure and covered with a mesh cloth. An orange tree was placed in the center of the field cage to simulate a natural environment. The test was performed with sexually mature flies of four cohorts, flies of thre differents ages (8–10 days old) (all flies emerged on the same date) of each cohort, three replicates each (*n* = 3), for a total of 36 experimental units for each treatment.

Mating of the males fed with the Mb^®^ enriched with *P. rettgeri* was compared to that of the males fed on the Mb^®^ alone. The males were marked in the upper thorax with non-toxic vinyl paint (Vinci de Mexico, S.A. de C.V., Mexico City), with different colors denoting the different treatments (Meza-Hernández and Díaz-Fleischer, 2006). For each cage, 25 males from each group (treatment and control) were introduced into the field ca ges, with 30 females introduced 15 min later. Thus, there was a total of 80 insects per cage every day. The observation time lasted from 6:30 am to 10:00 am, which corresponds to the period of maximum sexual activity for *A. obliqua* (Aluja et al., 2009), and mating pairs were removed from the field cage for scoring the treatments.

### Attractiveness Capture Test in Field Cage

This test was performed in field cages, as described above, using sexually mature flies of 8–10 days of age. Three Multilure^®^ traps were distributed randomly in the branches of the tree within the field cage. The traps were adapted into the inside of the lid with a basket made of tulle mesh and, within these baskets, 10 males from each treatment were confined, including a trap containing males feeding on the Mb^®^ enriched with *P. rettgeri*, another containing males feeding on Mb^®^ food alone and an empty third trap included as a control. At the base of the traps was a diluted solution of water and neutral soap in which to capture the visiting females. After 15 min, 30 females were released into the cage field in order to capture the sexually mature females attracted by the call of the males in the Multilure^®^ traps. The observation time was from 6:30 am to 10:00 am, and the number of females captured by each treatment was scored. The test was performed with sexually mature flies of four cohorts, flies of three differents ages (8–10 days old) (all flies emerged on the same date) of each cohort, three replicates each (*n* = 3), for a total of 36 experimental units for each treatment.

### Identification of the Pheromone Components

The sex pheromone was collected for two consecutive days using sexually mature males of 8 and 9 days of age after emerging from the same batch. Four batches of flies were used. Collection was performed at room temperature (25°C) in the Chemical Ecology Laboratory of ECOSUR. We used 10 males from each treatment, which were placed in 300 ml flasks that were completely sealed to prevent escape of the volatile of interest. The conditioned) needle of the Solid-phase microextraction (SPME) syringe was introduced into the flask to expose the polydimethylsiloxane/divinylbenzene (PDMS/DVB, Supelco, Bellefonte, PA, United States) fiber in order to collect the volatiles emitted by the males. Collection was conducted from 7:00 am to 10:00 am, covering the period of calling in the *A. obliqua* male. A total of 11 replicates were performed for each age and treatment.

Analysis of the volatiles was performed on a gas chromatograph coupled to a mass spectrometer (GC-MS) (CP-3800 Varian, Palo Alto, CA, United States) using a fused silica capillary column VF-5MS (30 m × 0.25 mm ID Varian, United States). The samples were injected into “splitless” mode. The carrier gas, high-purity helium, was used at a constant flow of 1 ml min^–1^. The temperature was programmed from 50°C for 1 min, increasing at 15°C min^–1^ up to 280°C, which was maintained for 10 min. The mass spectrometer was operated by electron impact (EI) at 70 eV. The transfer line was kept at a constant temperature of 280°C, while the ionization source was at 180°C. Exploration of the mass spectra was performed over a range of m/z 40–350 in SIM mode. The collected volatiles were identified by comparing the retention time and NIST-92 and NIST-98 mass spectrum databases. The area under each peak was determined, and this was the variable analyzed in order to assess the effect of diet on the sex pheromone components.

### Statistical Analysis

Statistical analysis of all data was performed using R software (R version 3.0.3) (R Core Team, 2018). Shapiro-Wilk normality test, Bartlett test of homogeneity of variances indicated that percentages of matings showed normality distribution and homogeneous variances. The data of matings and caught flies were analyzed by the Mixed-Effect model to determine the effect of the cohort and age as random factors, later the same data were analñyzed by a Generalized Linear Model (GLMs) considering a Poison (random) and log distribution. The data for volatile sex pheromone components were subjected to a *t*-test for unequal variances (α = 0.05).

## Results

### Bacteria Identification

One isolated colony, which presented 99.6% 16S *rRNA* gene sequence similarity with *P. rettgeri* (access number: NR042413) from the National Center for Biotechology Information (NCBI) gene bank, was selected. The bacterial identification with API (BioMerieux, Hazelwood, Mo) produced a >98% accuracy for *P. rettgeri* (Code API 20E: 027431157). This coincides with the comparison of the isolated 16S *rRNA* gene with the reference sequences deposited in NCBI.

### Sexual Competitiveness Tests in the Field Cage

**FIGURE 1:**
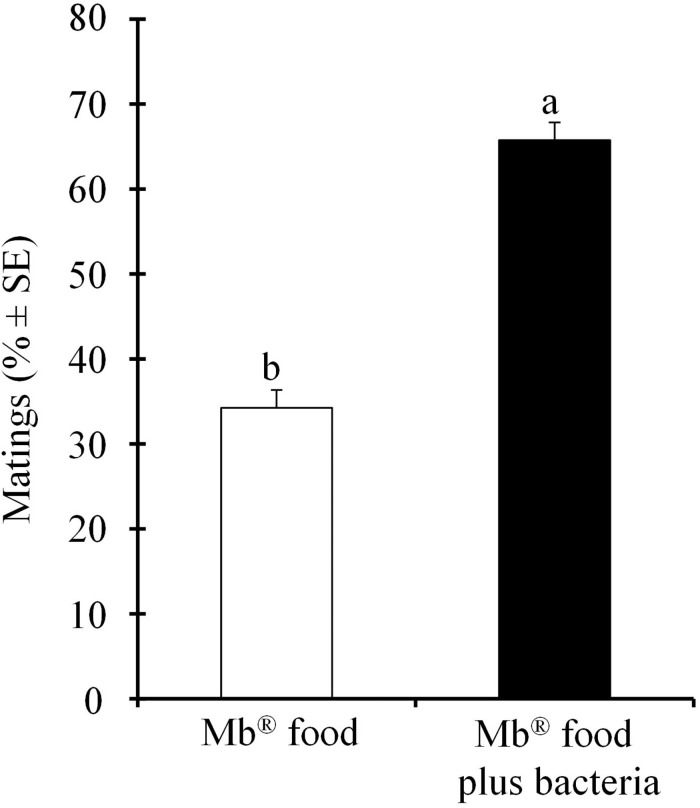
Mean percentages (± SE) of the number of mating *Anastrepha obliqua* males fed on Mb^®^ enriched with *Providencia rettgeri*, compared to the males fed on the Mb^®^ diet alone, in field cages.

We found that the number of mating *A. obliqua* males that were fed with Mb^®^ food enriched with *P. rettgeri* increased significantly (χ^2^ = 367.36; df = 1; *P* < 0.001) compared to those fed on the Mb^®^ food alone (Figure 1).

### Attractiveness Capture Test in the Field Cage

**FIGURE 2:**
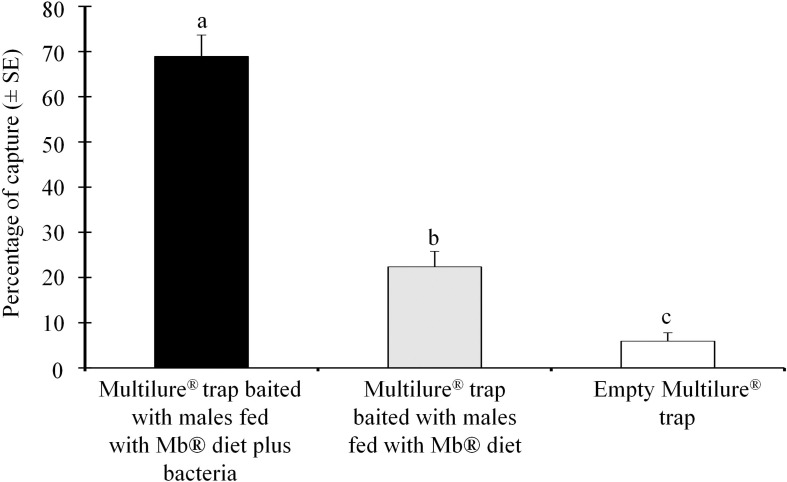
Mean percentages (± SE) of the number of female *Anastrepha obliqua* captured in Multilure traps baited with males fed on the Mb^®^ enriched with *Providencia rettgeri*, compared with the those on the Mb^®^ diet alone as a control.

We found that the Multilure traps baited with the males feeding on the Mb^®^ enriched with *P. rettgeri* captured significantly (χ^2^ = 2423.32; df = 1; *P* < 0.001) more females than the Multilure traps baited with the Mb^®^ food only (Figure 2). The empty (unbaited) Multilure traps captured the lowest number of females (Figure 2).

### Identification of the Pheromone Components

**FIGURE 3:**
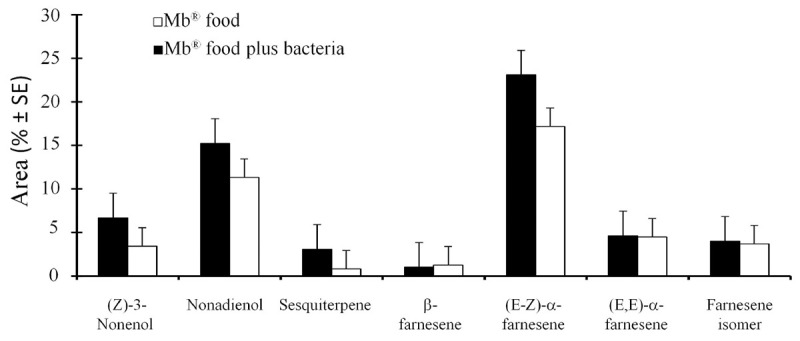
Mean percentages (± SE) of the quantity of volatiles released by the *A. obliqua* males fed on the Mb^®^ enriched with *Providencia rettgeri*, and by the males fed on the Mb^®^ diet alone.

Analysis of the sex pheromone components by GC-MS showed that the *A. obliqua* males fed on the Mb^®^ food enriched with *P. rettgeri* and the males fed only on Mb^®^ released seven compounds that were identified as (*Z*)-3-nonenol (*t* = 0.63; df = 14; *P* = 0.55), nonadienol (*t* = 0.36; df = 14; *P* = 0.73), sesquiterpene (*t* = 0.97; df = 14; *P* = 0.35), β-farnesene (*t* = 0.38; df = 14; *P* = 0.71), (E-Z)-α-farnesene (*t* = 0.56; df = 14; *P* = 0.58), (E,E)-α-farnesene (*t* = 0.07; df = 14; *P* = 0.94) and a farnesene isomer (*t* = 0.18; df = 14; *P* = 0.84) (Figure 3), which showed no significant difference in the proportion of pheromone components released between the two treatments.

## Discussion

This study produced three important findings: The first indicated that *A. obliqua* males from the mass-rearing colony fed on the Mb^®^ food enriched with *P. rettgeri* under field cage conditions presented increased mating compared to the males fed on the Mb^®^ food alone. The second finding showed that a higher number of females were captured with the Multilure traps baited with males fed on the Mb^®^ plus *P. rettgeri* diet. The third finding demonstrates that the *A. obliqua* males that were fed on the Mb^®^ and Mb^®^ enriched with *P. rettgeri* diets released the same compounds during their sexual calling and there was no significant difference between the quantities of each compound released by the males treated with the bacterium and by the control males. These results suggest that the Mb^®^ food enriched with *P. rettgeri* served to improve the sexual competitiveness of the *A. obliqua* males from the laboratory under field cage conditions. Similar results under laboratory conditions have been reported by Gómez-Alonso (2013), who determined the mating competitiveness of sterile *A. obliqua* and *A. serpentina* males fed on diets enriched with autogenous bacteria, using cages of 30 × 40 × 30 cm. The autogenous bacteria are present in the gut of the wild fruit flies and belong to the *Enterobacteriaceae* family (Behar et al., 2008; Ben-Yosef et al., 2008, 2010; Lauzon et al., 2009; Ben-Ami et al., 2010; Yuval et al., 2013).

Some of the functions attributed to these microorganisms include biosynthesis of nutrients that are minimally available or unavailable in the food (Cohen, 2004), such as essential amino acids, sugars and vitamins (Girolami, 1983). A few microorganisms have the capacity to fix the atmospheric nitrogen used by the organism *in vivo* in order to construct proteins involved in reproductive activity, i.e., *Citrobacter freundii, Enterobacter agglomerans, Desulfovibrio* spp., *Klebsiella* spp. and *Enterobacter* spp. (Nardi et al., 2002; Behar et al., 2005). *Providencia rettgeri* is a bacterium that is found in the gut of *A. ludens* (Kuzina et al., 2001) and was recently found in the gut of wild *A. obliqua* (Gómez-Alonso, 2013). The effects on the number of mating males and the capture of females in *A. obliqua* adults could be attributed to the essential amino acids, sugar or vitamins biosynthesized by the bacteria that are un available in the diet, or perhaps the *P. rettgeri* provided in the diet did not successfully establish in the gut and its contribution was merely as a nutrient source.

In this study, the volatile compounds found during sexual calling in *A. obliqua* coincide with those reported by López-Guillén et al. (2008, 2011) and Chacón-Benavente et al. (2013). Although a significant difference was not found, we observed that the release of (Z)-3-nonenol, nonadienol, sesquiterpene and (E-Z)-α-farnesene consistently increased when the males were fed with food enriched with *P. rettgeri*. In this way, Juárez et al. (2019) reported ten volatile compounds identified in *Anastrepha fraterculus* males, and they were significantly affected by the diet. They found that sugar fed males were significantly affected in all compounds released with exception of limonene, suspensolide, E, β-ocimene and unknown compound. In addition, in sugar + protein fed males, the authors not found differences (Juárez et al., 2019). Our results are different with those of Juárez et al. (2019), possibly by the lower number of replicates used in the volatile collection.

The hypothesis that food affects attraction, and therefore capture, is in accordance with Sharon et al. (2010), who affirmed that the symbiotic bacteria present in the gut of *Drosophila melanogaster* influence the production of cuticle hydrocarbons, which in turn modify the mating choice. This could explain the observed preference for mating with their counterparts that present the same type of gut bacteria, but does not explain why the females of A. obliqua that were fed with the food enriched with *P. rettgeri* were captured in greater numbers in the traps baited with males that had been fed the same diet. The diet determine the gut bacteria, and this in turn affects major cuticular hydrocarbons (Sharon et al., 2010), that according with Silk et al. (2017) enhance the responses to sex pheromone, as observed for the spruce budworm, *Choristoneura fumiferana*. The analysis of chemical signals depends on understanding the relationship between social and/or environmental context and the expression of different chemical combinations (Gershman et al., 2014). Although CHCs are not volatile, they can be broken into volatile fragments by reacting with environmental agents (Hatano et al., 2020), which could be used to produce a wide range of compounds as by-products of physiological processes and its regulation during the communication (Gershman et al., 2014).

In summary, in this study, we found that male *A. obliqua* adults that had been fed with the Mb^®^ food enriched with *P. rettgeri* presented significantly increased mating and captured more females in the field cages, suggesting that this diet acts to improve the sexual competitiveness of the males and can be used in the SIT.

## Data Availability Statement

The datasets generated for this study are available on request to the corresponding author.

## Author Contributions

EH and MA-M designed the experiment. LR-R, EH, and MA-M performed the experimental work. CV performed molecular identification of the bacteria. JT and EM designed the experimental, analyzed the data, and wrote the manuscript. All authors contributed to the article and approved the submitted version.

## Conflict of Interest

The authors declare that the research was conducted in the absence of any commercial or financial relationships that could be construed as a potential conflict of interest.
