# Supplementary material for: Attractiveness and Sexual Competitiveness of Anastrepha obliqua Males (Diptera: Tephritidae) Fed on a Diet Enriched With Providencia rettgeri
Source: Front Microbiol. 2020 Jul 28;11:1777. doi: 10.3389/fmicb.2020.01777 (PMC7509840; doi:10.3389/fmicb.2020.01777)
Supplement: Supplementary file 1 [file Table_1.DOCX]

Data

| Treatment | Cohort | Age | Replicate | Matings |
| --- | --- | --- | --- | --- |
| Control | 1 | 8 | 1 | 42.86 |
| Control | 1 | 8 | 2 | 42.86 |
| Control | 1 | 8 | 3 | 14.29 |
| Control | 1 | 9 | 1 | 44.44 |
| Control | 1 | 9 | 2 | 44.44 |
| Control | 1 | 9 | 3 | 50.00 |
| Control | 1 | 10 | 1 | 50.00 |
| Control | 1 | 10 | 2 | 54.55 |
| Control | 1 | 10 | 3 | 14.29 |
| Control | 2 | 8 | 1 | 40.00 |
| Control | 2 | 8 | 2 | 55.56 |
| Control | 2 | 8 | 3 | 26.67 |
| Control | 2 | 9 | 1 | 45.00 |
| Control | 2 | 9 | 2 | 41.18 |
| Control | 2 | 9 | 3 | 37.50 |
| Control | 2 | 10 | 1 | 30.77 |
| Control | 2 | 10 | 2 | 38.89 |
| Control | 2 | 10 | 3 | 37.50 |
| Control | 3 | 8 | 1 | 14.29 |
| Control | 3 | 8 | 2 | 27.27 |
| Control | 3 | 8 | 3 | 37.50 |
| Control | 3 | 9 | 1 | 38.46 |
| Control | 3 | 9 | 2 | 45.45 |
| Control | 3 | 9 | 3 | 11.11 |
| Control | 3 | 10 | 1 | 30.00 |
| Control | 3 | 10 | 2 | 9.09 |
| Control | 3 | 10 | 3 | 25.00 |
| Control | 4 | 8 | 1 | 30.00 |
| Control | 4 | 8 | 2 | 40.00 |
| Control | 4 | 8 | 3 | 22.22 |
| Control | 4 | 9 | 1 | 18.18 |
| Control | 4 | 9 | 2 | 33.33 |
| Control | 4 | 9 | 3 | 42.86 |
| Control | 4 | 10 | 1 | 20.00 |
| Control | 4 | 10 | 2 | 37.50 |
| Control | 4 | 10 | 3 | 40.00 |
| Enriched | 1 | 8 | 1 | 57.14 |
| Enriched | 1 | 8 | 2 | 57.14 |
| Enriched | 1 | 8 | 3 | 85.71 |
| Enriched | 1 | 9 | 1 | 55.56 |
| Enriched | 1 | 9 | 2 | 55.56 |
| Enriched | 1 | 9 | 3 | 50.00 |
| Enriched | 1 | 10 | 1 | 50.00 |
| Enriched | 1 | 10 | 2 | 45.45 |
| Enriched | 1 | 10 | 3 | 85.71 |
| Enriched | 2 | 8 | 1 | 60.00 |
| Enriched | 2 | 8 | 2 | 44.44 |
| Enriched | 2 | 8 | 3 | 73.33 |
| Enriched | 2 | 9 | 1 | 55.00 |
| Enriched | 2 | 9 | 2 | 58.82 |
| Enriched | 2 | 9 | 3 | 62.50 |
| Enriched | 2 | 10 | 1 | 69.23 |
| Enriched | 2 | 10 | 2 | 61.11 |
| Enriched | 2 | 10 | 3 | 62.50 |
| Enriched | 3 | 8 | 1 | 85.71 |
| Enriched | 3 | 8 | 2 | 72.73 |
| Enriched | 3 | 8 | 3 | 62.50 |
| Enriched | 3 | 9 | 1 | 61.54 |
| Enriched | 3 | 9 | 2 | 54.55 |
| Enriched | 3 | 9 | 3 | 88.89 |
| Enriched | 3 | 10 | 1 | 70.00 |
| Enriched | 3 | 10 | 2 | 90.91 |
| Enriched | 3 | 10 | 3 | 75.00 |
| Enriched | 4 | 8 | 1 | 70.00 |
| Enriched | 4 | 8 | 2 | 60.00 |
| Enriched | 4 | 8 | 3 | 77.78 |
| Enriched | 4 | 9 | 1 | 81.82 |
| Enriched | 4 | 9 | 2 | 66.67 |
| Enriched | 4 | 9 | 3 | 57.14 |
| Enriched | 4 | 10 | 1 | 80.00 |
| Enriched | 4 | 10 | 2 | 62.50 |
| Enriched | 4 | 10 | 3 | 60.00 |

Capture test

| Treatment | Cohorte | Age | Replicate | Captures |
| --- | --- | --- | --- | --- |
| Control | 1 | 8 | 1 | 50.00 |
| Control | 1 | 8 | 2 | 50.00 |
| Control | 1 | 8 | 3 | 20.00 |
| Control | 1 | 9 | 1 | 100.00 |
| Control | 1 | 9 | 2 | 16.67 |
| Control | 1 | 9 | 3 | 25.00 |
| Control | 1 | 10 | 1 | 40.00 |
| Control | 1 | 10 | 2 | 0.00 |
| Control | 1 | 10 | 3 | 25.00 |
| Control | 2 | 8 | 1 | 0.00 |
| Control | 2 | 8 | 2 | 0.00 |
| Control | 2 | 8 | 3 | 0.00 |
| Control | 2 | 9 | 1 | 25.00 |
| Control | 2 | 9 | 2 | 16.67 |
| Control | 2 | 9 | 3 | 25.00 |
| Control | 2 | 10 | 1 | 50.00 |
| Control | 2 | 10 | 2 | 33.33 |
| Control | 2 | 10 | 3 | 16.67 |
| Control | 3 | 8 | 1 | 33.33 |
| Control | 3 | 8 | 2 | 28.57 |
| Control | 3 | 8 | 3 | 40.00 |
| Control | 3 | 9 | 1 | 33.33 |
| Control | 3 | 9 | 2 | 0.00 |
| Control | 3 | 9 | 3 | 25.00 |
| Control | 3 | 10 | 1 | 16.67 |
| Control | 3 | 10 | 2 | 20.00 |
| Control | 3 | 10 | 3 | 0.00 |
| Control | 4 | 8 | 1 | 0.00 |
| Control | 4 | 8 | 2 | 16.67 |
| Control | 4 | 8 | 3 | 33.33 |
| Control | 4 | 9 | 1 | 20.00 |
| Control | 4 | 9 | 2 | 25.00 |
| Control | 4 | 9 | 3 | 0.00 |
| Control | 4 | 10 | 1 | 0.00 |
| Control | 4 | 10 | 2 | 20.00 |
| Control | 4 | 10 | 3 | 0.00 |
| Enriched | 1 | 8 | 1 | 25.00 |
| Enriched | 1 | 8 | 2 | 0.00 |
| Enriched | 1 | 8 | 3 | 80.00 |
| Enriched | 1 | 9 | 1 | 0.00 |
| Enriched | 1 | 9 | 2 | 66.67 |
| Enriched | 1 | 9 | 3 | 75.00 |
| Enriched | 1 | 10 | 1 | 40.00 |
| Enriched | 1 | 10 | 2 | 0.00 |
| Enriched | 1 | 10 | 3 | 75.00 |
| Enriched | 2 | 8 | 1 | 100.00 |
| Enriched | 2 | 8 | 2 | 100.00 |
| Enriched | 2 | 8 | 3 | 100.00 |
| Enriched | 2 | 9 | 1 | 75.00 |
| Enriched | 2 | 9 | 2 | 66.67 |
| Enriched | 2 | 9 | 3 | 75.00 |
| Enriched | 2 | 10 | 1 | 50.00 |
| Enriched | 2 | 10 | 2 | 66.67 |
| Enriched | 2 | 10 | 3 | 66.67 |
| Enriched | 3 | 8 | 1 | 66.67 |
| Enriched | 3 | 8 | 2 | 71.43 |
| Enriched | 3 | 8 | 3 | 50.00 |
| Enriched | 3 | 9 | 1 | 66.67 |
| Enriched | 3 | 9 | 2 | 100.00 |
| Enriched | 3 | 9 | 3 | 50.00 |
| Enriched | 3 | 10 | 1 | 83.33 |
| Enriched | 3 | 10 | 2 | 80.00 |
| Enriched | 3 | 10 | 3 | 100.00 |
| Enriched | 4 | 8 | 1 | 100.00 |
| Enriched | 4 | 8 | 2 | 66.67 |
| Enriched | 4 | 8 | 3 | 50.00 |
| Enriched | 4 | 9 | 1 | 80.00 |
| Enriched | 4 | 9 | 2 | 75.00 |
| Enriched | 4 | 9 | 3 | 100.00 |
| Enriched | 4 | 10 | 1 | 100.00 |
| Enriched | 4 | 10 | 2 | 80.00 |
| Enriched | 4 | 10 | 3 | 100.00 |
| Empty_traps | 1 | 8 | 1 | 25.00 |
| Empty_traps | 1 | 8 | 2 | 50.00 |
| Empty_traps | 1 | 8 | 3 | 0.00 |
| Empty_traps | 1 | 9 | 1 | 0.00 |
| Empty_traps | 1 | 9 | 2 | 16.67 |
| Empty_traps | 1 | 9 | 3 | 0.00 |
| Empty_traps | 1 | 10 | 1 | 20.00 |
| Empty_traps | 1 | 10 | 2 | 0.00 |
| Empty_traps | 1 | 10 | 3 | 0.00 |
| Empty_traps | 2 | 8 | 1 | 0.00 |
| Empty_traps | 2 | 8 | 2 | 0.00 |
| Empty_traps | 2 | 8 | 3 | 0.00 |
| Empty_traps | 2 | 9 | 1 | 0.00 |
| Empty_traps | 2 | 9 | 2 | 16.67 |
| Empty_traps | 2 | 9 | 3 | 0.00 |
| Empty_traps | 2 | 10 | 1 | 0.00 |
| Empty_traps | 2 | 10 | 2 | 0.00 |
| Empty_traps | 2 | 10 | 3 | 16.67 |
| Empty_traps | 3 | 8 | 1 | 0.00 |
| Empty_traps | 3 | 8 | 2 | 0.00 |
| Empty_traps | 3 | 8 | 3 | 10.00 |
| Empty_traps | 3 | 9 | 1 | 0.00 |
| Empty_traps | 3 | 9 | 2 | 0.00 |
| Empty_traps | 3 | 9 | 3 | 25.00 |
| Empty_traps | 3 | 10 | 1 | 0.00 |
| Empty_traps | 3 | 10 | 2 | 0.00 |
| Empty_traps | 3 | 10 | 3 | 0.00 |
| Empty_traps | 4 | 8 | 1 | 0.00 |
| Empty_traps | 4 | 8 | 2 | 16.67 |
| Empty_traps | 4 | 8 | 3 | 16.67 |
| Empty_traps | 4 | 9 | 1 | 0.00 |
| Empty_traps | 4 | 9 | 2 | 0.00 |
| Empty_traps | 4 | 9 | 3 | 0.00 |
| Empty_traps | 4 | 10 | 1 | 0.00 |
| Empty_traps | 4 | 10 | 2 | 0.00 |
| Empty_traps | 4 | 10 | 3 | 0.00 |

Volatiles

| alimento | dia | nonenol | nonadienol | 3 | 4 | (E-z)-a-farneseno | (E,E)-a-farneseno | 7 |
| --- | --- | --- | --- | --- | --- | --- | --- | --- |
| MB+Bacteria | 8 | 14631908 | 30420572 | 6818815 | 1140014 | 30420572 | 5927136 | 5219595 |
| MB+Bacteria | 8 | 1924316 | 4054997 | 476464 | 778231 | 13276793 | 2741287 | 1659629 |
| MB+Bacteria | 8 | 115524 | 1616606 | 111595 | 112315 | 1834653 | 439230 | 476453 |
| MB+Bacteria | 8 | 0 | 70044 | 68914 | 57261 | 2144537 | 410112 | 413022 |
| MB+Bacteria | 9 | 417903 | 974891 | 738596 | 111526 | 3918419 | 914103 | 757678 |
| MB+Bacteria | 9 | 1928928 | 3795415 | 372377 | 508478 | 6054270 | 1139128 | 1184997 |
| MB+Bacteria | 9 | 0 | 2019603 | 93334 | 119064 | 5228209 | 1027593 | 1034383 |
| MB+Bacteria | 9 | 0 | 371299 | 101275 | 141818 | 2723570 | 584503 | 701541 |
| MB | 8 | 3190135 | 12461903 | 682709 | 1016003 | 12190848 | 2541574 | 2069211 |
| MB | 8 | 3728916 | 8557611 | 620273 | 856756 | 7873390 | 1645577 | 1588441 |
| MB | 8 | 0 | 229211 | 126747 | 124415 | 647341 | 2167087 | 168078 |
| MB | 8 | 0 | 1428557 | 66818 | 60641 | 111704 | 216783 | 181416 |
| MB | 9 | 1483969 | 4299519 | 516790 | 1021502 | 10501439 | 2176228 | 2958128 |
| MB | 9 | 1242256 | 1243953 | 217125 | 318905 | 5581023 | 1086962 | 759606 |
| MB | 9 | 102167 | 3572297 | 332 | 109088 | 7490347 | 1566012 | 1407016 |
| MB | 9 | 0 | 345220 | 98809 | 97686 | 4381279 | 1362398 | 1357272 |
